# Imaging the Future: Diagnosing Treatable Neurometabolic Disorders in Children

**DOI:** 10.7759/cureus.90124

**Published:** 2025-08-14

**Authors:** Ishani Reddy Eda, Rajoo Ramachandran, Veena M Joseph, Sahithi Marreddy, Arikila Mounika

**Affiliations:** 1 Department of Radiology, Sri Ramachandra Institute of Higher Education and Research, Chennai, IND; 2 Department of Radiology, Velindre Cancer Centre, Cardiff, GBR; 3 Radiology, Sindhu Hospitals, Hyderabad, IND

**Keywords:** cerebral creatine deficiency syndrome, gamt deficiency, hypermanganesemia, magnetic resonance spectroscopy, mri brain, pediatric neurometabolic disorders, slc19a3, slc25a19, slc39a14, thiamine metabolism dysfunction syndrome

## Abstract

Inborn errors of metabolism are a prevalent cause of pediatric neurological abnormalities, often resulting from enzyme deficiencies that disrupt metabolic pathways. Understanding the radiological manifestations of these disorders is critical for timely diagnosis and therapy. This study aimed to elucidate the magnetic resonance imaging (MRI) brain characteristics and magnetic resonance spectroscopy (MRS) findings of specific treatable pediatric neurometabolic disorders through clinical vignettes, thereby enhancing diagnostic accuracy and informing therapeutic approaches. The study includes four cases with varying presentations, all confirmed by genetic testing, and utilized magnetic resonance imaging and spectroscopy to identify characteristic features. The first case, diagnosed as thiamine metabolism dysfunction syndrome type 4, exhibits bilateral corpus striatum T2 hyperintensities with diffusion restriction on MRI brain, increased lactate peak, and reduced N-acetylaspartate (NAA) on magnetic resonance spectroscopy (MRS), and SLC25A19 gene mutation on genetic analysis - features consistent with biotin-thiamine-responsive basal ganglia disease. The second case, identified as thiamine metabolism dysfunction syndrome type 2, reveals T2 hyperintensities in the bilateral corpus striatum, medial thalami, and cerebellar white matter, along with restricted diffusion, reduced NAA, and an inverted lactate doublet on MRS, with genetic analysis confirming an SLC19A3 mutation, also falling under the spectrum of biotin-thiamine-responsive basal ganglia disease. The third case, representing cerebral creatine deficiency syndrome type 2, demonstrates bilateral symmetric T2 hyperintensities in the globus pallidus and central tegmental tracts, a characteristic absence of a creatine peak on MRS, and a confirmed guanidomethyl transferase (GAMT) deficiency gene mutation. The fourth case, diagnosed as hypermanganesemia with dystonia type 2, is characterized by T1 hyperintensity and T2 hypointensity involving the bilateral globus pallidi, substantia nigra, and dentate nuclei, with genetic testing revealing an SLC39A14 mutation. The study emphasizes the importance of MR imaging and spectroscopy in identifying pediatric neurometabolic diseases that can be treated.

## Introduction

Inborn errors of metabolism (IEMs) are conditions arising from disruptions in particular metabolic processes or abnormalities in enzymes, their cofactors, or transporters, leading to substrate buildup or inhibition of product production [1,2]. Around 25% of incidents develop during the newborn phase, characterized by severe encephalopathy, resulting in a dismal prognosis if not managed. The role of IEMs in causing neurological symptoms in pediatric patients, such as developmental delay, seizures, movement impairments (e.g., dystonia, chorea), hypotonia, and cognitive impairments or neurodegeneration, is becoming more well acknowledged. Neuroimaging, especially magnetic resonance imaging (MRI) of the brain, has emerged as an effective tool for the initial assessment of children with suspected neurometabolic disorders. Specific patterns of brain involvement, such as symmetrical signal changes in the deep gray nuclei (e.g., basal ganglia, thalami) or white matter anomalies, frequently lead toward particular metabolic etiologies [3]. Magnetic resonance spectroscopy (MRS) enhances diagnostic precision by enabling non-invasive assessment of brain metabolites, including N-acetylaspartate (NAA), creatine, choline, lactate, and myoinositol. Early detection of these imaging characteristics is crucial for timely diagnosis, initiation of treatment, and genetic counselling. Accordingly, the collection of clinical, biochemical, and radiological data is essential for optimal patient care. This case series evaluates four genetically validated and radiologically characterized pediatric neurometabolic diseases, each exhibiting a unique imaging phenotype and available targeted therapy.

## Case presentation

Case 1: thiamine metabolism dysfunction syndrome type 4

**Figure 1:**
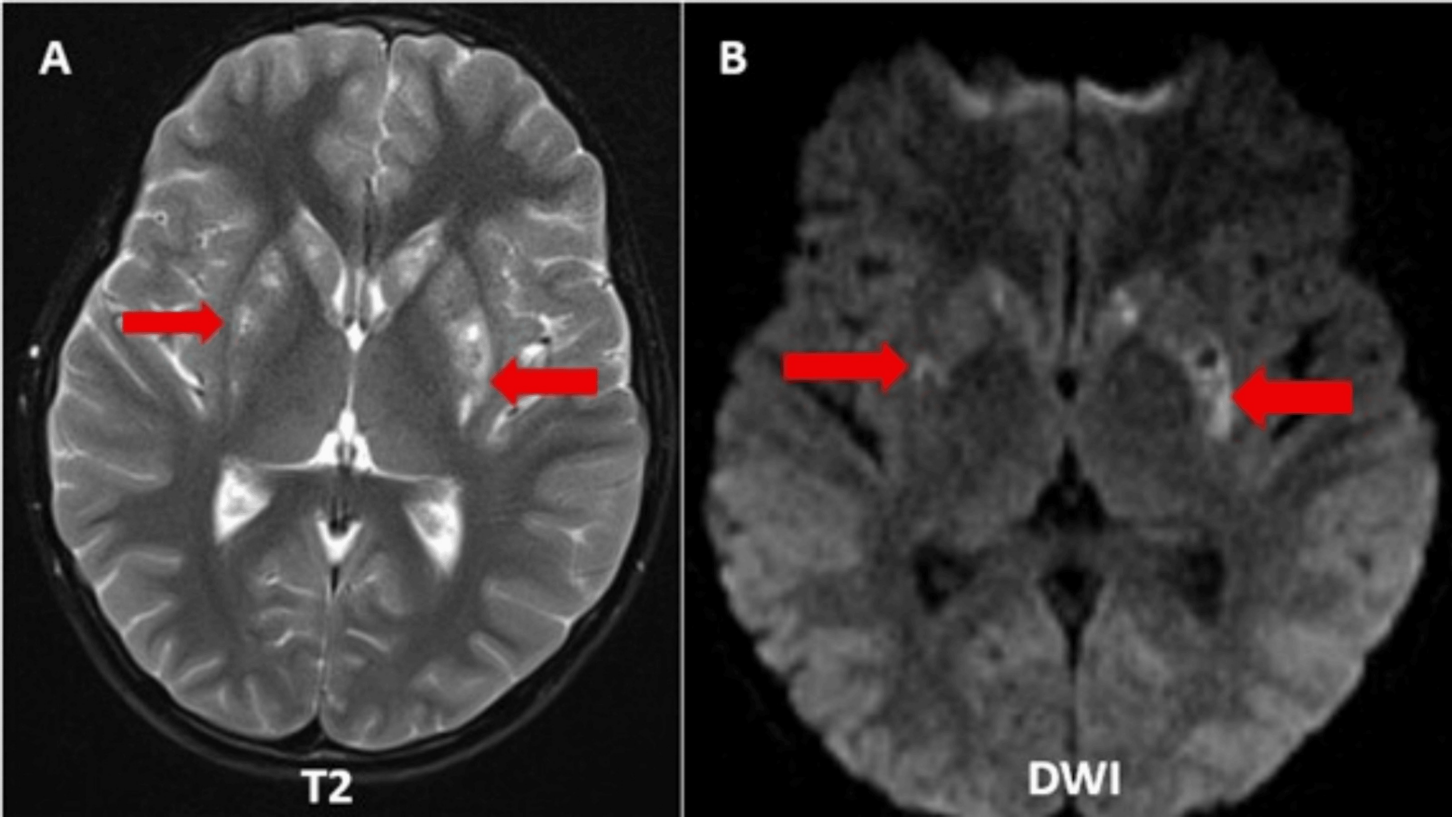
Axial sections of MRI brain (bilateral corpus striatum). (A) Multiple T2 hyperintense foci in bilateral corpus striatum (red arrows). (B) Restricted diffusion in the same areas (red arrows). MRI: magnetic resonance imaging; DWI: diffusion-weighted imaging

**Figure 2:**
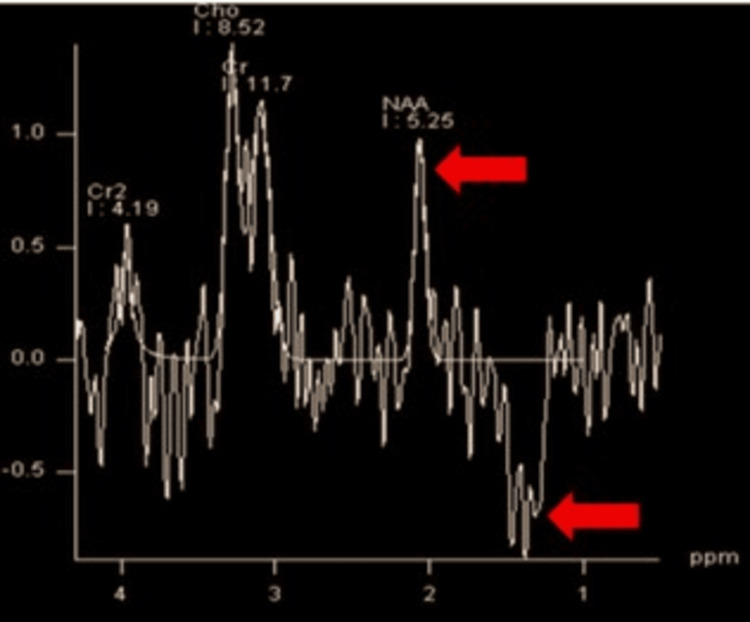
MRS at 135 ms shows decreased NAA and inverted peak of lactate doublet (red arrows). MRS: magnetic resonance spectroscopy; NAA: N-acetylaspartate

This is a case of an eight-year-old male with complaints of dystonia with no significant family or prenatal history of a related neurological condition. The MRI brain demonstrates multiple foci of T2 hyperintensities in the bilateral corpus striatum (Figure 1A), accompanied by diffusion restriction on diffusion-weighted imaging (DWI) (Figure 1B). Magnetic resonance spectroscopy (MRS) at 135 ms shows an increased lactate peak (inverted doublet) and diminished concentrations of N-acetylaspartate (NAA) (Figure 2).

The results are in line with the radiological characteristics commonly observed in biotin-thiamine-responsive basal ganglia disease, with the globus pallidus appearing spared and the thalamus and cerebellum showing varying involvement. Genetic testing verifies a mutation in the SLC25A19 gene, with alterations detected in exons 5 and 6, indicative of probable compound heterozygosity. The diagnosis of thiamine metabolism dysfunction syndrome type 4 (which is progressive polyneuropathy type), an autosomal recessive disorder, is established. The therapeutic management involves high-dose dietary supplementation of biotin and thiamine, with early initiation being essential to prevent irreversible neurological damage.

Case 2: thiamine metabolism dysfunction syndrome type 2

**Figure 3:**
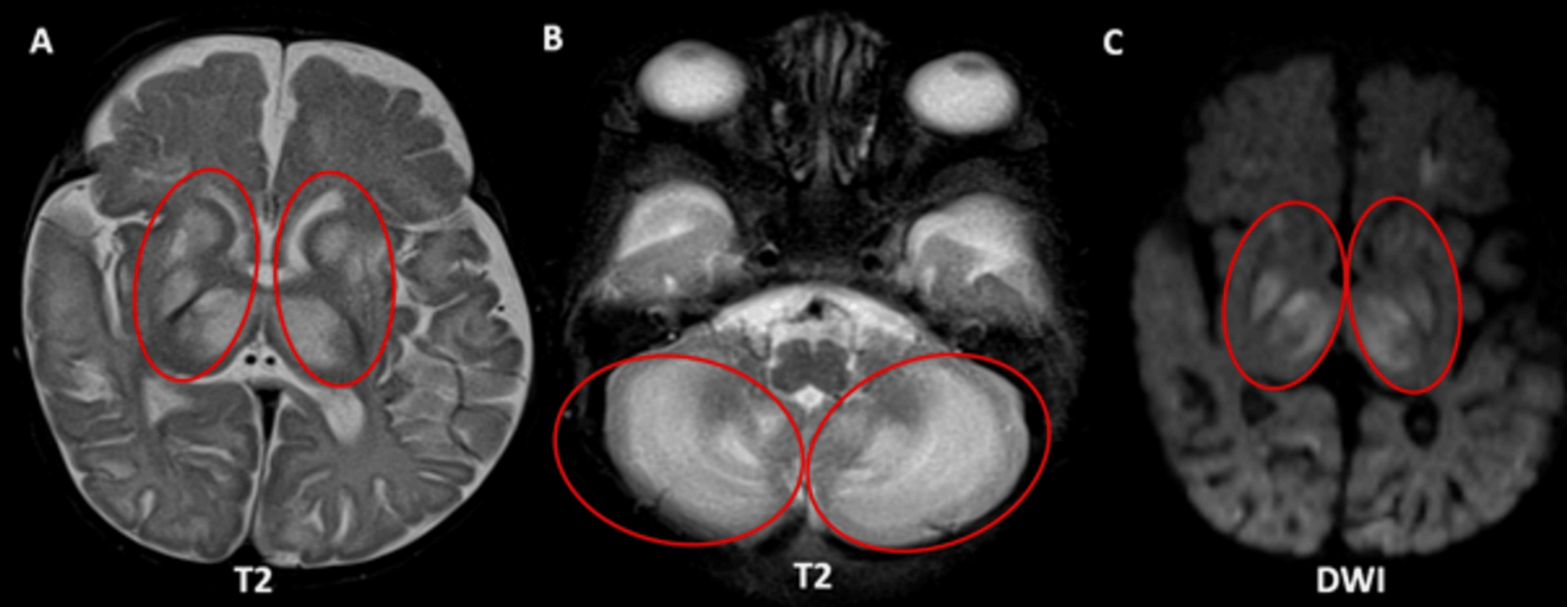
Axial sections of MRI brain (corpus striatum, medial thalami, cerebellar white matter). (A) Multiple areas of T2 hyperintensities in the bilateral corpus striatum and medial thalami (red circles). (B) Multiple areas of T2 hyperintensities in the cerebellar white matter (red circles). (C) Corresponding areas of abnormal restricted diffusion on DWI (red circles). MRI: magnetic resonance imaging; DWI: diffusion-weighted imaging

**Figure 4:**
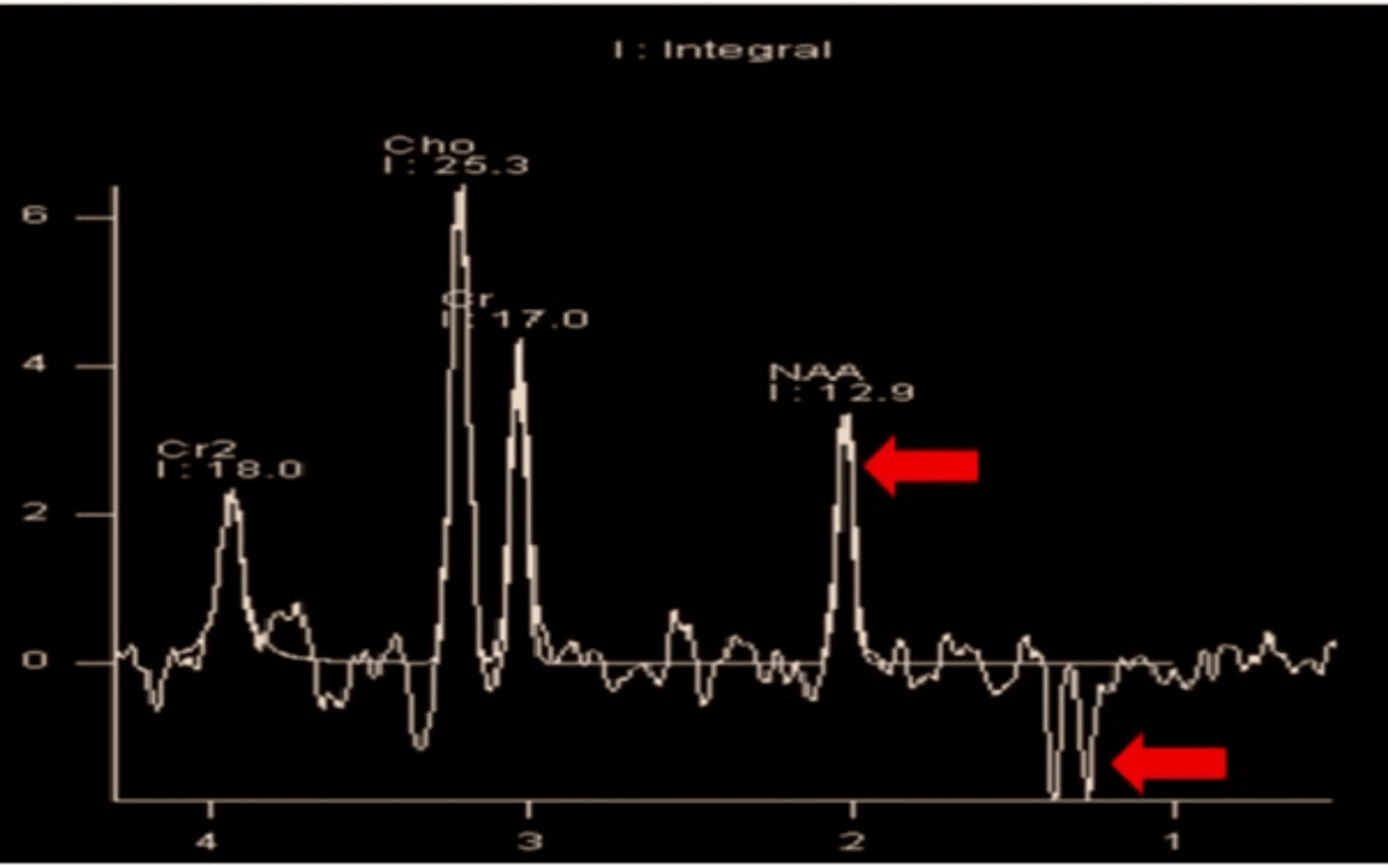
MRS at 135 ms demonstrates diminished NAA levels and an inverted lactate doublet signal (red arrows). MRS: magnetic resonance spectroscopy; NAA: N-acetylaspartate

A three-month-old child exhibited atypical motions and global developmental delay with a history of a sibling death. MRI brain demonstrates multiple areas of T2 hyperintensities in the bilateral corpus striatum, medial thalami, and cerebellar white matter, along with restricted diffusion on diffusion-weighted imaging (DWI) (Figures 3A-3C). Magnetic resonance spectroscopy (MRS) performed at 135 ms reveals reduced N-acetylaspartate (NAA) levels and an inverted lactate doublet peak, implying compromised brain energy metabolism (Figure 4).

The radiological characteristics strongly indicate a metabolic disease impacting energy-dependent areas of the brain. Genetic testing verifies a homozygous mutation in exon 3 of the SLC19A3 gene, indicating a diagnosis of thiamine metabolism dysfunction syndrome type 2, also falling under the spectrum of biotin-thiamine-responsive basal ganglia disease. This autosomal recessive disorder results from a deficiency in the brain transport of biotin and thiamine, causing neuronal energy loss. The therapeutic management involves high-dose dietary supplementation of biotin and thiamine.

Case 3: cerebral creatine deficiency syndrome type 2

**Figure 5:**
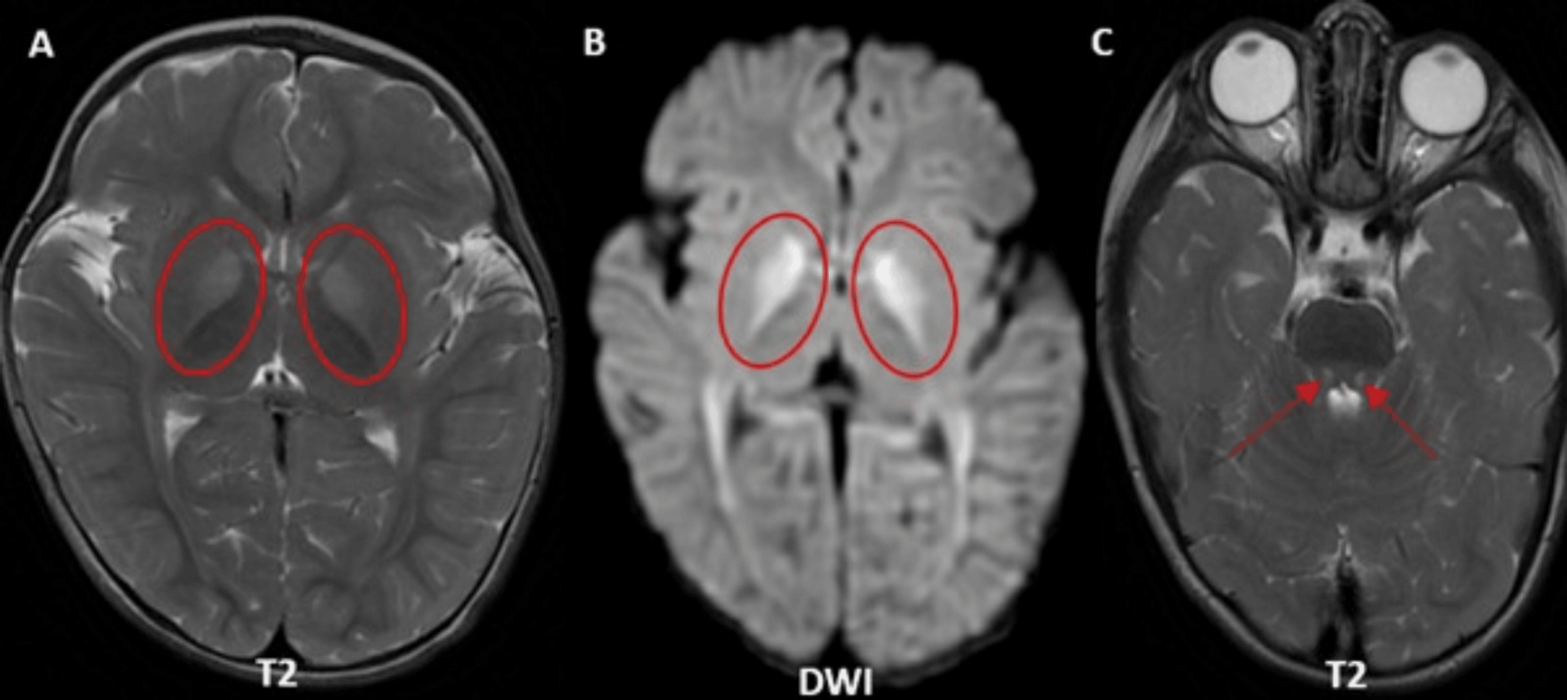
Axial sections of MRI brain (globus pallidi and central tegmental tracts). (A) Bilateral symmetrical T2 hyperintensities in the globus pallidi (red circles). (B) Corresponding restricted diffusion on DWI in the globus pallidi (red circles). (C) Bilateral symmetrical T2 hyperintensities in central tegmental tracts (red arrows). MRI: magnetic resonance imaging; DWI: diffusion-weighted imaging

**Figure 6:**
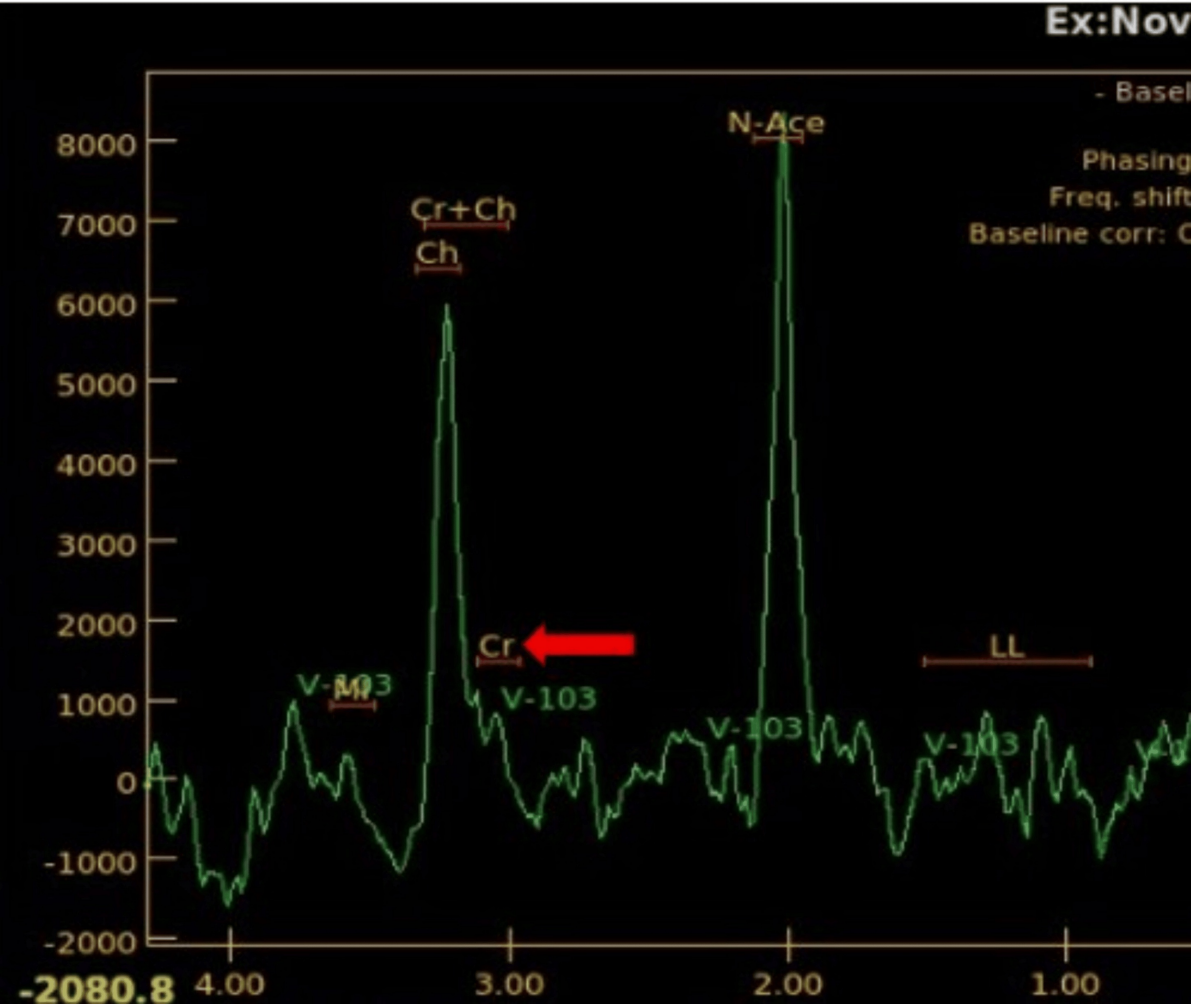
MRS at 135 ms showing absent creatine peak (red arrow). MRS: magnetic resonance spectroscopy

This is a case of a four-year-old child presenting with developmental delay. MRI brain reveals bilateral symmetrical T2 hyperintensities in the globus pallidus and central tegmental tract, accompanied by limited diffusion on diffusion-weighted imaging (DWI) (Figure 5A-5C). Magnetic resonance spectroscopy (MRS) at 135 ms reveals a missing creatine peak at 3 ppm (parts per million), a definitive indicator of creatine insufficiency (Figure 6).

Genetic testing identified a homozygous mutation in the guanidinoacetate methyltransferase (GAMT) gene, particularly within intron 2, hence validating a diagnosis of GAMT deficiency. Cerebral creatine deficiency syndrome type 2 (CCDS-2), an autosomal recessive condition, is among the three types of genetic anomalies that encompass CCDS, which impact creatine synthesis or transport. Out of these, GAMT deficiency is a manageable variant. The syndrome leads to compromised creatine production, resulting in neurological impairments. The principal intervention for GAMT insufficiency is oral creatine monohydrate supplementation, which aids in restoring brain creatine levels and may enhance neurological outcomes over time.

Case 4: hypermanganesemia with dystonia type 2

**Figure 7:**
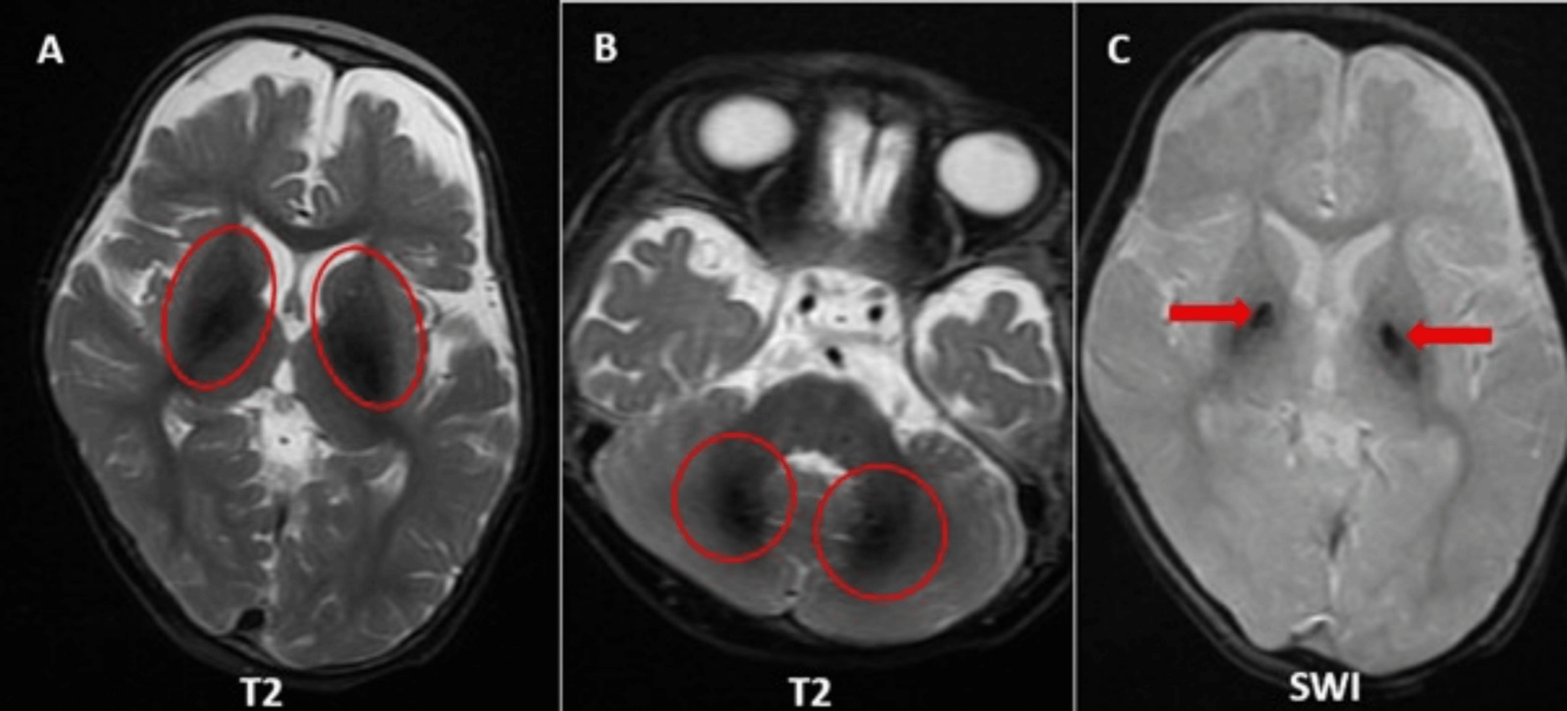
Axial sections of MRI brain (T2-weighted MRIs and SWI). (A) T2 hypointensity in the bilateral globus pallidi (red circles). (B) T2 hypointensity in the bilateral dentate nuclei (red circles). (C) Increased paramagnetic susceptibility in SWI sequences (red arrows). MRI: magnetic resonance imaging; SWI: susceptibility-weighted imaging

**Figure 8:**
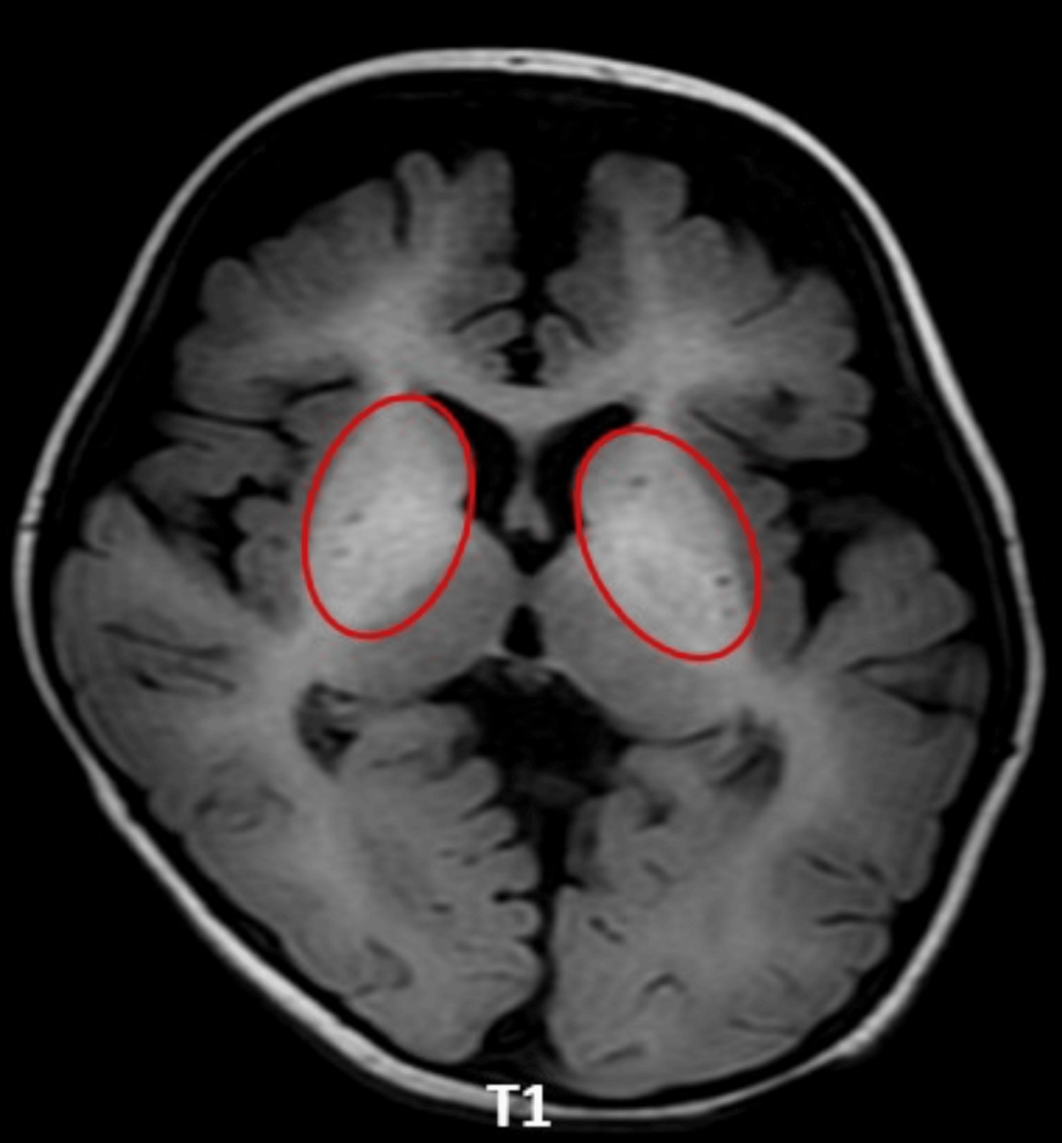
Axial T1 sequence of MRI brain shows hyperintensity of the white matter (red circles). MRI: magnetic resonance imaging

This is a case of a three-month-old male infant with hypotonia and suspected neuroregression. A progressive decline in motor abilities raised concerns for a neurodegenerative process. MRI brain shows T2 hypointensity in the bilateral globus pallidi, substantia nigra, and dentate nuclei (Figures 7A, 7B), as well as blooming on susceptibility-weighted imaging (SWI) (Figure 7C). Increased T1 hyperintensity is seen in the white matter (Figure 8).

The imaging results indicate atypical metal deposition, predominantly correlating with manganese buildup. Genetic testing indicates a homozygous mutation in the SLC39A14 gene on exon 6, which is crucial for manganese transport and homeostasis. A diagnosis of hypermanganesemia with dystonia type 2, an autosomal recessive neurological disorder, was obtained based on clinical symptoms, imaging data, and genetic analysis. Early diagnosis is crucial as chelation therapy may offer benefit if initiated promptly.

## Discussion

This case series demonstrates the crucial importance of neuroimaging, including MRI brain and magnetic resonance spectroscopy (MRS), in the early identification and intervention of pediatric neurometabolic disorders. All four instances exhibited a manageable metabolic disease, with neuroimaging serving a pivotal function in clinical suspicion, diagnostic validation, and continuous care.

In this case series, cases 1 and 2 demonstrated the classical imaging and clinical characteristics of biotin-thiamine-responsive basal ganglia disease, including symmetrical T2 hyperintensities in the corpus striatum, diffusion restriction, and distinctive MRS findings (elevated lactate and diminished NAA). These findings are consistent with the hallmark radiological patterns described by Biswas et al., who emphasized the utility of clinico-radiological phenotyping in guiding early diagnosis in childhood neurometabolic disorders. The involvement of energy-dependent systems, such as the basal ganglia and thalami, shown in our patients, was recognized as a diagnostic indicator within their framework [4].

Kassem et al. assessed neurometabolic diseases in pediatric patients by brain MRI. All instances exhibited bilateral caudate lesions with involvement of the putamen and preservation of the globus pallidus. Irregular signals were detected in the mesencephalon, cortex, and thalami (80%), deep white matter (53%), and cerebellum (13.3%). Following treatment, signal anomalies cleared, with the exception of enduring necrosis in the caudate and putamen, even in asymptomatic individuals [5]. Our case 2 also had a sibling with a history of early death, suggesting a potential genetic illness.

Similarly, Alfadhel et al. documented 18 genetically validated cases with mutations in SLC19A3, accompanied by consistent radiological observations and positive responses to early supplementation. Our study confirmed genetic alterations in SLC19A3 and SLC25A19, highlighting the molecular heterogeneity while maintaining radiological similarities among these individuals. Our studies corroborate Alfadhel’s claim that early identification and intervention substantially modify the illness trajectory, frequently resulting in enhanced neurological outcomes [6].

In our case series, case 3 was diagnosed as having guanidinoacetate methyltransferase (GAMT) deficiency, a rare but treatable subtype of cerebral creatine deficiency syndromes (CCDS). The patient demonstrated developmental delay and presented distinctive neuroimaging findings, including symmetrical T2 hyperintensities in the globus pallidus and central tegmental tracts, as well as an absence of a creatine peak at 3 ppm on MR spectroscopy (MRS). These radiological features align closely with those reported in the study by Yoganathan et al., who emphasized the diagnostic significance of absent creatine peaks on MRS in confirming CCDS [7].

Yoganathan et al. highlighted that the lack of a creatine peak is both diagnostic and beneficial for monitoring treatment efficacy. In this study, the imaging findings were crucial for diagnosis and initiating treatment with oral creatine monohydrate, which is consistent with their suggested protocol. This underscores the significance of MRS in both identifying and managing CCDS cases.

Additionally, in their systematic review, Rohilla et al. described a systematic diagnostic approach to inborn neurometabolic disorders and pointed out that neurodevelopmental symptoms can be substantially reversed by early detection of GAMT deficiency. Their classification of GAMT deficiency as one of the rare metabolic disorders with significant treatment promise if detected early was supported by our study, in which MRI, MRS, and genetic confirmation were used to establish the diagnosis [1].

A mutation in the SLC39A14 gene verified the diagnosis of hypermanganesemia with dystonia type 2 (HMNDYT2) in case 4 of our study. Indicative of manganese buildup, MRI results showed T1 hyperintensity and T2 hypointensity in the globus pallidus, substantia nigra, and dentate nuclei. These imaging characteristics are consistent with those reported by Madhubala et al., who observed comparable MRI patterns in a female newborn with HMNDYT2 who was nine months old [8]. Patients with HMNDYT2 have demonstrated clinical improvement after receiving chelation therapy, specifically calcium disodium edetate. According to Marti-Sanchez et al., chelation therapy used early on improved symptoms and decreased manganese levels [9].

## Conclusions

This case series concludes by highlighting the vital diagnostic function of MRI brain and MRS in diagnosing pediatric neurometabolic disorders. When combined with clinical and genetic profiles, characteristic radiological patterns can expedite diagnosis and treatment choices. Improving outcomes in this susceptible population requires raising clinician awareness of these imaging characteristics.

Radiologists should be familiar with the pattern of involvement, their radiologic appearance, and clinical significance for optimal patient management. Timely recognition of the treatable causes of inborn errors is critical, as early intervention can markedly improve the patient's prognosis and lead to significant neurological recovery, particularly in conditions where effective treatment options are available, as illustrated in the aforementioned cases.
